# Automatic detection of genomic regions with informative epigenetic patterns

**DOI:** 10.1186/s12864-018-5286-5

**Published:** 2018-11-28

**Authors:** Florencio Pazos, Adrian Garcia-Moreno, Juan C. Oliveros

**Affiliations:** National Center for Biotechnology (CNB-CSIC), c/ Darwin, 3, 28049 Madrid, Spain

**Keywords:** Epigenomics, Epigenetics, Gene transcription regulation

## Abstract

**Background:**

Epigenetic phenomena are crucial for explaining the phenotypic plasticity seen in the cells of different tissues, developmental stages and diseases, all holding the same DNA sequence. As technology is allowing to retrieve epigenetic information in a genome-wide fashion, massive epigenomic datasets are being accumulated in public repositories. New approaches are required to mine those data to extract useful knowledge. We present here an automatic approach for detecting genomic regions with epigenetic variation patterns across samples related to a grouping of these samples, as a way of detecting regions functionally associated to the phenomenon behind the classification.

**Results:**

We show that the regions automatically detected by the method in the whole human genome associated to three different classifications of a set of epigenomes (cancer vs. healthy, brain vs. other organs, and fetal vs. adult tissues) are enriched in genes associated to these processes.

**Conclusions:**

The method is fully automatic and can exhaustively scan the whole human genome at any resolution using large collections of epigenomes as input, although it also produces good results with small datasets. Consequently, it will be valuable for obtaining functional information from the incoming epigenomic information as it continues to accumulate.

**Electronic supplementary material:**

The online version of this article (10.1186/s12864-018-5286-5) contains supplementary material, which is available to authorized users.

## Background

The expression of the information encoded in the genome is determined not only by its DNA sequence but by many other factors that affect the complex process of gene expression [1]. These factors include reversible modifications in the DNA and histones that affect gene expression without altering the nucleotide sequence of the DNA, for example by changing the folding state or accessibility of the chromatin. These are called epigenetic modifications, and “epigenomics” is the high-throughput characterization of these modifications at a genomic scale [2, 3]. Epigenetic control of gene expression has been shown to be crucial in processes such as development and cell differentiation [4, 5], as well as in diseases [6, 7]. In the case of diseases, epigenetic mechanisms are gaining increasing attention since they could explain the influence of environmental factors in a pathological state [8].

**Fig. 1:**
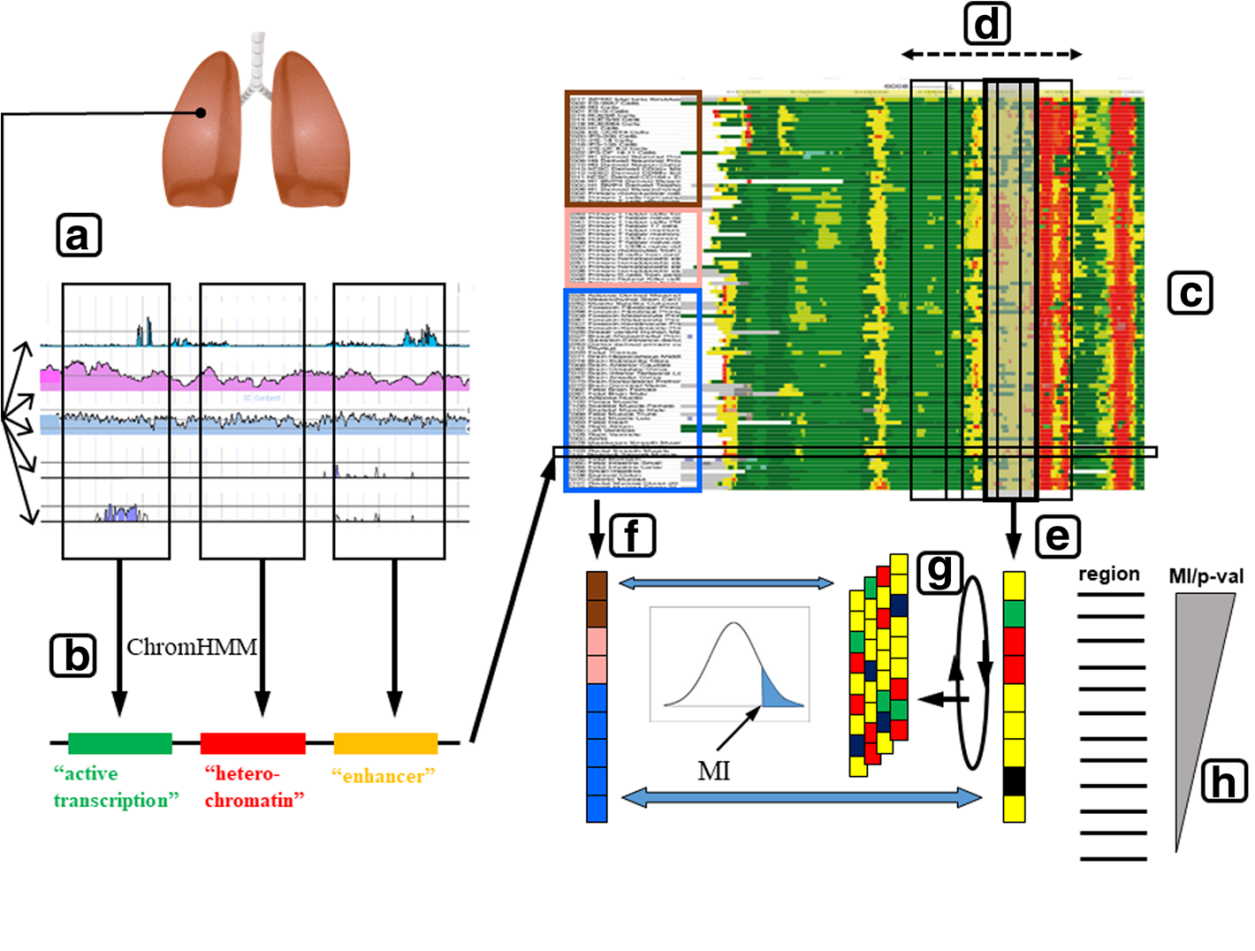
Schema of the methodology used for detecting genome segments with an epigenetic pattern resembling a sample classification. For a given sample, different epigenomic markers are quantified in a genome-wide fashion (**a**). For a given region of the genome, all these markers are collapsed into a single epigenetic state (colors) using ChromHMM (**b**). This is done for hundreds of different samples (**c**). These samples can be classified according with different criteria (into three groups in this example: brown, pink and blue). The epigenetic pattern of a given segment of the genome (**e**) is compared with an equivalent pattern representing this sample classification (**f**) using a “mutual information” based approach. One of the patterns is shuffled thousands of times in order to generate a null distribution of MI scores from where to extract a p-value for the MI score of the segment of interest (**g**). The process is repeated for all other windows in the human genome (**d**). The genome segments with the highest MI values and significant p-values are taken as those related to the sample classification (**h**). Lung clipart source: Wikimedia Commons (http://commons.wikimedia.org/)

Using assays based on deep sequencing, it is possible to detect in a genome-wide fashion different epigenetic modifications (i.e. covalent modifications of DNA and histones), as well as other proxies of the epigenetic state of a genome segment (e.g. mRNA expression, nucleosome occupancy), for a given sample (Fig. 1). Recently, different consortia at national and international levels were assembled with the objective of obtaining this genome-wide epigenomic information for hundreds of samples (“epigenomes”), including different organs, developmental states and cancer lines [3]. Examples of such initiatives are ENCODE [9], BLUEPRINT [10] and Roadmap Epigenomics [11]. This massive epigenomic information is starting to be mined in a similar way as genomic data. For example, it is being used, alone or in combination with other evidences, to reconstruct the 3D structure of the chromatin [12–14], detect functionally related genes [15], interpreting the results of “genome-wide association studies” (GWAS) [16] and non-coding variants in general [17]. This genome-wide epigenetic information is also being used for epigenomic-GWAS (EGWAS) [18], to stablish links between epigenetic variation in certain loci and diseases. Recently, it was also used to look for regions whose quantitative epigenetic marks change between two sets of samples, an strategy implemented, for example, in the ChromSwitch package [19].

From a practical point of view, all those different indicators of epigenetic state for a given genome segment can be collapsed into a single “epigenetic state” easier to be interpreted (e.g. “active transcription”, “enhancer”, …) (Fig. 1). This is done by training machine-learning systems with segments of known epigenetic state and their corresponding profiles of epigenetic proxies. Once trained, these methods are used to infer the state for a given region based on its raw epigenetic profiles. Examples of these methodologies are ChromHMM [20] and Segway [21].

These datasets of chromatin states are conceptually similar to protein or DNA multiple sequence alignments (MSAs): the epigenomes would be the homologous proteins and the genome positions the residues (Fig. 1). Indeed, methodologies originally developed for protein and DNA alignments are being adapted to these datasets: e.g. Epilogos (https://epilogos.altiusinstitute.org/) for generating state-logos (equivalent to sequence logos), and ChromDet [22] for the unsupervised classification of epigenomes and the detection of chromatin regions associated to that classification (CDRs: “chromatin determining regions”). These are equivalent to the “specificity determining residues” (SDPs) in protein MSAs: residues differentially conserved in the subfamilies of the MSA and hence responsible for their functional specificity [23–25].

Nevertheless, such a rich source of information is still scarcely explored, compared with the thousands of approaches available for studying, for example, DNA sequences (“genomics”). Apart from providing interesting information on particular genes and cellular processes, the large scale analysis of these epigenomic datasets might provide systemic information. Moreover, while all studies report a large global epigenetic variation contributing to cell differentiation, locating where the epigenetic changes responsible for it exactly occur is still a challenge.

In this work we propose a methodology for mining the growing epigenomic data in the search for functional genomic regions. This approach automatically detects genomic regions whose epigenetic profile across a set of samples is related to a user-provide classification of these samples. For the regions detected in this way that contain coding genes, we show that these are involved in the biological processes associated to the sample classification. We also show that the method is robust to the lack of input epigenomic information and can work with a reduced number of epigenomes, although it is fast enough for handling the expected increasing number of epigenomes in the future.

## Methods

In order to find genomic regions whose inter-sample epigenomic variation correlates with a given classification of these, we used the epigenomic datasets compiled by the Roamap Epigenomics Consortium [11]. We downloaded the 127 “consolidated epigenomes”, for which the chromatin states are given in a 15-state vocabulary, from https://egg2.wustl.edu/roadmap/web_portal/chr_state_learning.html

These 127 epigenomes include different tissues, primary cells, cancer cell lines, different developmental stages, etc. For each epigenome, detailed information on different proxies of the epigenetic state at 1 bp resolution is provided, such as histone acetylation, DNA methylation, DNase accessibility or RNA-seq expression (“a” in Fig. 1). For each 200 bp genomic region, the ChromHMM method [20] was used to collapse all these experimental epigenetic proxies into a single “epigenetic state” with 15 possible values (“b” in Fig. 1). From the URL above, we downloaded the. BED files with the mnemonics indicating the chromatin states along the 127 epigenomes at that 200 bp resolution (“c” in Fig. 1). The original 15-state vocabulary can be simplified by grouping states representing similar epigenetic behavior. Apart from the original 15 states, for this work we also tried two reductions (to 5 and 2 states –i.e. “active” vs. “inactive”-) previously used by [22].

In order to scan the human genome for segments with a given pattern of epigenetic changes, we use a sliding window of a given length (200 bp or larger) and move it at different steps along the whole genome (“d” in Fig. 1). The epigenetic state for that window in a given epigenome is taken as that with the highest frequency among all 200 bp segments within the window. A minimum frequency of 80% is required. Otherwise, the “undefined” state is assigned to that window. So, for a given window, we end up in a vector (epigenetic profile) where each component represents an epigenome and its value is the epigenetic state at that particular genomic region (with 15, 5 or 2 possible states, depending on the vocabulary used, plus “undefined”) (“e” in Fig. 1). An equivalent vector is constructed representing the classification of the samples in a given number of groups (“f” in Fig. 1). The similarity between these two vectors is quantified by their Mutual Information. The mutual information between the group classification vector (*G*) and the epigenetic profile of the *i*_*th*_ segment (*S*_*i*_) is calculated as:

$$ MI\left(G:{S}_i\right)=\sum \limits_{k=1}^n\sum \limits_{l=1}^mP\left({G}_k,{S}_{il}\right)\bullet {\log}_2\frac{P\left({G}_k,{S}_{il}\right)}{P\left({G}_k\right)\bullet P\left({S}_{il}\right)} $$

Where the sums run for all possible groups in the sample classification (*n*) and all possible epigenetic states (*m*). *P*(*G*_*k*_) is the frequency of group *k*, *P*(*S*_*il*_) is the frequency of the epigenetic state *l* in window *i*, and *P*(*G*_*k*_, *S*_*il*_) the frequency of group *k* matching state *l*. State “undefined” is ignored for this calculation, and the terms of the sums where either *P*(*G*_*k*_) or *P*(*S*_*il*_) are 0 are skipped. Consequently, this parameter has a high value for a window when its epigenetic profile correlates with the sample classification. That is, all samples of the same group tend to present the same epigenetic state, and the other way around. In that case there is a relationship between the epigenetic states and the group classification.

To assess the significance of an observed MI value we shuffle the components of epigenetic profile vector of the corresponding window a number of times (10,000 in this work) and calculate the distribution of MI values of the sample vector against these random vectors. The composition of the window vector is preserved in this randomization, and only the positions of the labels are changed (“g” in Fig. 1). From this background distribution, a *p*-value for a given observed MI score is calculated as the fraction of randomized vectors resulting in higher or equal MI’s. These *p*-values are additionally adjusted for multiple testing using Benjamini-Hochberg’s FDR correction. So, at the end, for a given group classification, we end up with a list of all genome segments (windows) and their associated MI score and *p*-values (“h” in Fig. 1).

To summarize the epigenetic change pattern of a given window across a large number of samples classified in groups, we just take the majority state for each group.

To show the potential of this methodology to locate genomic regions associated to different phenomena, we applied it to three different classifications of subsets the 127 epigenomes:

- Brain vs. other tissues. We filtered out samples from cultured cells, cancer-derived and those annotated as “other”. Our final list contains 75 epigenomes from which 10 belong to the “brain” group (including different brain anatomical parts, male/female, fetal/adult, etc.)
- Cancer vs. normal. We took the five cancer epigenomes available and all samples of the corresponding healthy tissues. So, we do not include a sample of a given tissue if there is no cancer version for it. The final list contains 21 epigenomes (5 cancer).
- Fetal vs. other. We filtered out samples from cultured cells and cancer-derived. The final list contains 79 epigenomes, 13 of which belong to different fetal tissues.

These datasets are available as Additional file 1.

In order to interpret the large number of genome regions detected by this procedure when they contain annotated genes, we retrieve these genes with Bedtools [26]. For that we use the latest annotation available for the genome assembly our epigenomic data is based on (GRCh37.p13), downloaded from the NCBI ftp site. Any gene overlapping, totally or partially, with the genome segment of interest is retrieved.

To assess whether a collection of genes (coming from a set of genome segments of interest) is representing a particular biological process or processes we performed a “functional enrichment analysis”. This standard analysis looks for functional keywords/terms overrepresented in the set of genes of interest respect to a background (the whole human genome in this case). For this we used the topGO R package [27], fed with the Gene Ontology (GO) [28] annotations for the human genes downloaded from the NCBI. We chose the Fisher’s exact test implemented in this package and subsequently applied Benjamini-Hochberg FDR correction for multiple testing for quantifying the overrepresentation of a given GO term in a set of genes.

We evaluate the effect of the number available epigenomes on the quality of the results obtained for the “brain” dataset. In this dataset it is possible to automatically detect the enriched GO terms unambiguously related to “brain” (i.e. those containing keywords such as “brain”, “neuron”, “nerve”, “axon”, … and related), as a proxy for “positive” terms. For this dataset, we repeated the procedure described above randomly removing increasing proportions of the initial epigenomes (from 20 to 80%). Every removal was repeated 5 times and the results averaged. We quantify the performance of the method in the original dataset as well as in the reduced sets as the negative logarithm of the enrichment *p*-value for those “positive” GO biological processes. While this is far from being a real quantification of performance (e.g. many terms without these keywords, such as their parents, can also be positives) it allows us to compare its relative change in this down-sampling experiment.

## Results

We applied the procedure described in Methods to the three classification schemas in order to detect the genomic regions associated to the phenomena behind the classifications. In the three cases, we scanned the whole human genome with consecutive non-overlapping windows of length 5000 bp, applying the procedure described in Methods with a reduced vocabulary of 5 states, and took the segments with MI score of 0.3 or higher and a FDR-corrected *p*-value of 1E-4 or lower.

### Brain samples

In the case of the brain tissues, it is expected that the genomic regions detected are in some way related to the function and development of this organ, since they present a characteristic epigenetic behavior in this organ different from that in others. We found 2991 regions in the genome with MI score 0.3 or higher and a FDR-corrected p-value of 1E-4 or lower. Chromosome 1 has the highest number of these regions (296). The lists of regions (sorted by the MI score) for each chromosome are available as Additional file 2. The lists include links to a genome browser to inspect the genomic neighbourhood of each window, as well as their epigenetic profiles in comparison with the group classification (brain vs. others).

These regions overlap with 1030 different genes according with the annotation procedure followed. Two hundred forty-six regions (8%) do not overlap with any gene. The enrichment analysis of Gene Ontology terms clearly shows that these 1030 genes are enriched in biological processes related to neuronal development and functioning, such as “nervous system development”, “neurogenesis”, “generation of neurons”, “neuron differentiation”, … (Table 1). These genes are also enriched in Gene Ontology “cellular compartment” annotations (GO_CC) such as “neuron part”, “synapse”, “neuron projection”. The whole list of enriched terms is available in the Additional file 3, and a graphical summary of these, generated with REVIGO [29], in the Additional file 1: Figure S3.

**Table 1:** Enriched processes in the three datasets. For the brain dataset, only the top of the list is shown. The whole lists with additional information (e.g. genes in each process), are available as Additional file 3

	BRAIN			FETAL			CANCER	
GO.ID	Term	Fisher Test	GO.ID	Term	Fisher Test	GO.ID	Term	Fisher Test
GO:0007399	nervous system development	< 1e-30	GO:0007166	cell surface receptor signaling pathway	1.80E-05	GO:0000278	mitotic cell cycle	1.60E-04
GO:0022008	neurogenesis	6.50E-27	GO:0007167	enzyme linked receptor protein signaling...	1.50E-04	GO:0098912	membrane depolarization during atrial ca...	1.90E-04
GO:0031175	neuron projection development	7.90E-27	GO:0009966	regulation of signal transduction	1.90E-04	GO:0007049	cell cycle	2.60E-04
GO:0030182	neuron differentiation	1.80E-26	GO:0035272	exocrine system development	2.00E-04	GO:0060850	regulation of transcription involved in cell fate	3.70E-04
GO:0048699	generation of neurons	2.60E-26	GO:0001503	ossification	2.10E-04	GO:0006928	movement of cell or subcellular componen...	4.40E-04
GO:0048666	neuron development	7.00E-26	GO:0060605	tube lumen cavitation	3.00E-04	GO:0007018	microtubule-based movement	5.90E-04
GO:0048812	neuron projection morphogenesis	2.00E-23	GO:0060662	salivary gland cavitation	3.00E-04	GO:0086045	membrane depolarization during AV node c...	6.20E-04
GO:0048468	cell development	2.50E-23	GO:0023051	regulation of signaling	3.30E-04	GO:0007417	central nervous system development	9.50E-04
GO:0048731	system development	8.40E-22	GO:0016055	Wnt signaling pathway	4.20E-04			
GO:0048667	cell morphogenesis involved in neuron di...	2.40E-21	GO:0010646	regulation of cell communication	4.20E-04			
GO:0050808	synapse organization	2.40E-19	GO:0022008	neurogenesis	5.10E-04			
GO:0048856	anatomical structure development	5.70E-19	GO:0007169	transmembrane receptor protein tyrosine ...	5.20E-04			
GO:0030030	cell projection organization	1.10E-18	GO:0048699	generation of neurons	6.30E-04			
GO:0007267	cell-cell signaling	2.50E-17	GO:0023056	positive regulation of signaling	7.90E-04			
GO:0007275	multicellular organismal development	3.00E-17	GO:0060137	maternal process involved in parturition	8.40E-04			
GO:0048858	cell projection morphogenesis	4.40E-17	GO:2000794	regulation of epithelial cell proliferat...	8.40E-04			
GO:0007268	synaptic transmission	4.70E-17	GO:0007435	salivary gland morphogenesis	8.80E-04			
GO:0023052	signaling	5.70E-17	GO:0010647	positive regulation of cell communicatio...	9.50E-04			
GO:0032990	cell part morphogenesis	6.40E-17						
GO:0044700	single organism signaling	7.70E-17						
GO:0061564	axon development	9.80E-17						
GO:0000904	cell morphogenesis involved in different...	3.00E-16						
GO:0032502	developmental process	3.30E-16						
GO:0007409	axonogenesis	7.60E-16						
GO:0044767	single-organism developmental process	9.80E-16						
GO:0032989	cellular component morphogenesis	2.80E-15						
GO:0000902	cell morphogenesis	2.90E-15						
GO:0051960	regulation of nervous system development	5.30E-15						
GO:0050803	regulation of synapse structure or activ...	8.80E-15						
GO:0007154	cell communication	1.60E-14						
GO:0007416	synapse assembly	2.10E-14						
GO:0050804	modulation of synaptic transmission	7.00E-13						
GO:0044707	single-multicellular organism process	8.10E-13						

**Fig. 2:**
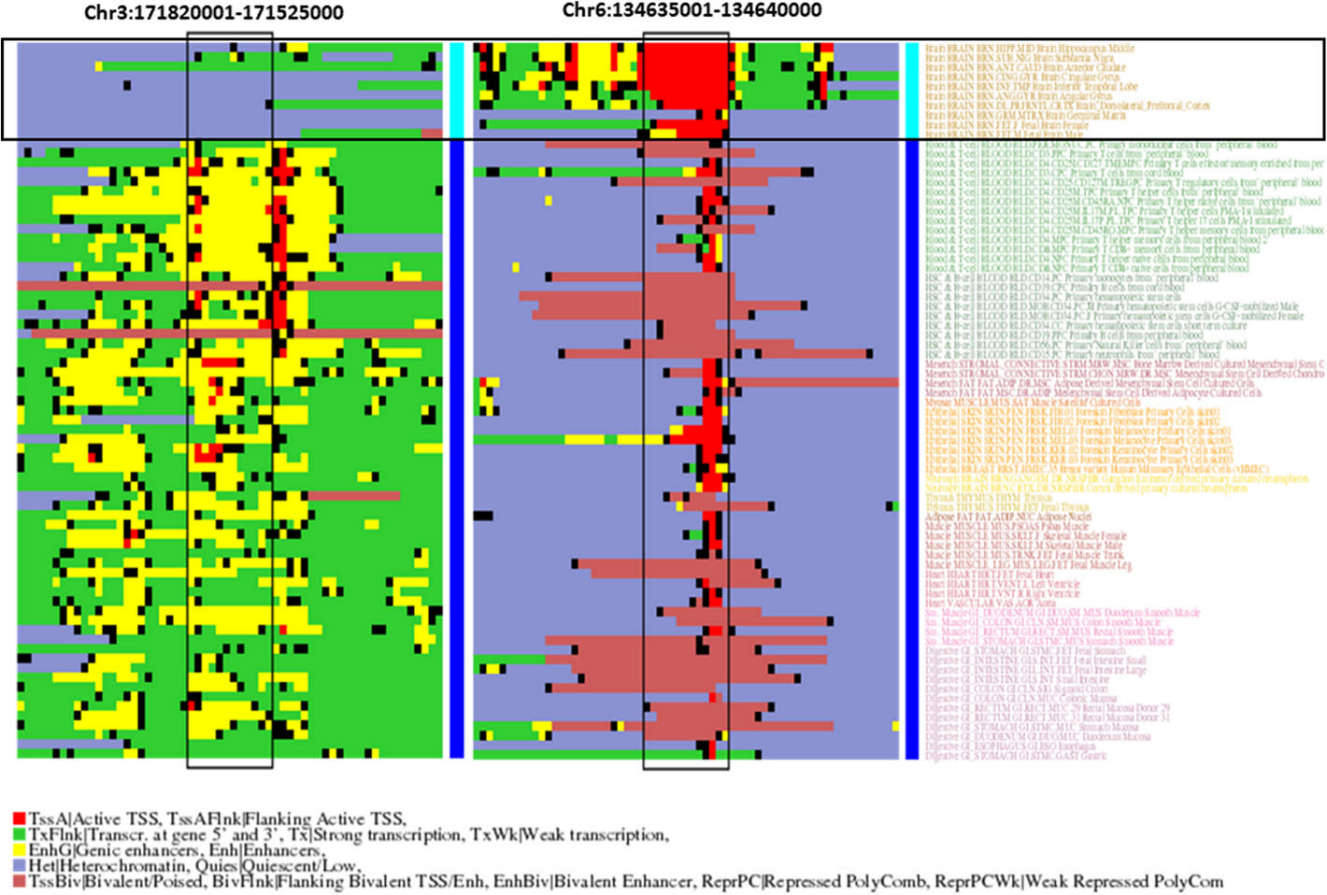
Example of two genomic regions with an epigenetic pattern related to brain. The epigenetic state of each 400 bp portion (small boxes) is indicated with colors (legend at the bottom). The two discussed regions (5000 bp windows) are highlighted, surrounded by their epigenomic neighborhood. The sample classification is indicated with light blue (brain) and dark blue (others). The brain samples are further highlighted with a box. The sample names are on the right, colored using the tissue-based color schema of the Roadmap Epigenomics Consortium

The region with the highest MI score is 134,635,001–134,640,000 in chromosome 6. The epigenetic profile of this region shows that it tends to be in “active transcription” state in the brain samples while heterochromatized/repressed in the others (Fig. 2). This region matches part of an intron and the last exon of gene SGK1, a kinase that plays a role in neuron excitability by activating a number of ion channels. The next region in the list (Chr12:49255001–49,260,000) has a similar epigenetic profile and overlaps with intronic and exonic regions of gene RND1, a Rho GTPase involved in axon extension. The third region with highest score (Chr16:56310001–56,315,000) has another epigenetic profile: it is in “enhancer” state in brain tissues while heterochromatized in others. It matches an intronic region of gene GNAO1, a G protein repressor, whose defects are a cause of early-onset epileptic encephalopathy. The fourth hit (Chr3:171820001–171,525,000) has an opposite epigenetic profile: inactive/heterochromatin in most brain tissues, and enhancer/weak transcription in the rest (Fig. 2). This window corresponds to an intronic region of gene FNDC3B, a fribonectin type III containing protein without obvious relationship with the brain. Nevertheless, the expression of that gene in the brain (from the GTEx project [30]) is much lower than in any other tissue (Additional file 1: Figure S1), in agreement with its epigenetic profile and pointing to a relationship with this organ.

As shown in Fig. 5, the two most frequent patterns of epigenetic change between brain samples and those of other tissues in these 2991 windows is transcription (brain) / heterochromatin (others), followed by transcription (b) / bivalent chromatin (o). The third pattern is the opposite (heterochromatin (b) / transcription (o), and the fourth pattern is enhancer (b) / heterochromatin (o). We performed the GO enrichment analysis described above for the windows within these different sets independently, to get insight into the molecular processes associated to these different epigenetic changes. In the following, we focus on the GO terms differentially enriched in these subsets (respect to those enriched in the whole set commented above). The two first patterns, which involve specific activation of transcription in brain samples, are related to neuron development and function (biological processes) and synapsis and ion channels (cellular compartments). The opposite pattern (silencing in brain) is related to “apoptosis”, “cell death” and similar terms (In GO:CC, the term differentially enriched for this pattern is “intracellular”). This points to a scenario where processes related to neuron development are “activated” in brain while those related to cell removal/turnover are “silenced”. For the fourth pattern, the enriched terms are related to nucleotide metabolic processes. The detailed results of this subset enrichment analysis are available as Additional file 4.

The accumulation of these high-MI 5000 bp windows in certain regions can also point to interesting sites. That is, even if none of these windows is at the top of the list, as the four we discussed above, the fact that they concentrate in a particular region of the genome could be informative. Probably that region would show up scanning with a larger window. The gene with the largest number of high-MI windows is CSMD2. Fifty out of the 2991 windows match this long gene. This gene is involved in the control of complement, and defects on it have been associated with schizophrenia. The next gene is CTNND2 (49 windows). This gene encodes an adhesive junction associated protein involved in brain and eye development, and it resides in a region of chromosome 5 deleted in the *Cri du Chat* syndrome, characterized by intellectual disability and microcephaly. The other genes with high concentration of informative windows are also related to brain in one way or another (data not shown).

We used this “brain” dataset to evaluate the effect of the number of available epigenomes on the performance of the method, since the “correctness” of the final set of GO:BP enriched terms is easier to assess automatically in this dataset (see Methods). Additional file 1: Figure S2 shows the variation of performance as we remove increasing proportions of the input epigenomes. It can be seen that the results are quite stable until we remove a large proportion of the epigenomes (60%), a point where it drops sharply. When 80% of the epigenomes are removed, we either ended up without any brain sample in the input set of epigenomes (i.e. no results are generated) or the results are bad and no brain-related GO:BP terms showed up as enriched.

### Fetal samples

We found 224 regions in the genome associated to this phenomenon with the procedure described above (Additional file 2), with chromosome 7 containing the largest number of them (38), followed by chromosome X (22).

These windows overlap with 130 annotated genes. 20% of them (44 regions) do not overlap with any gene. These genes are enriched in biological functions related to organism development and signal transduction, such as “regulation of signal transduction”, “neurogenesis”, “ossification” and morphogenesis of different organs (Table 1). There are no “cellular compartment” (CC) terms enriched with the cutoffs used. The whole list of enriched terms is available in the Additional file 3, and a graphical summary of these, generated with REVIGO [29], in the Additional file 1: Figure S4.

**Fig. 3:**
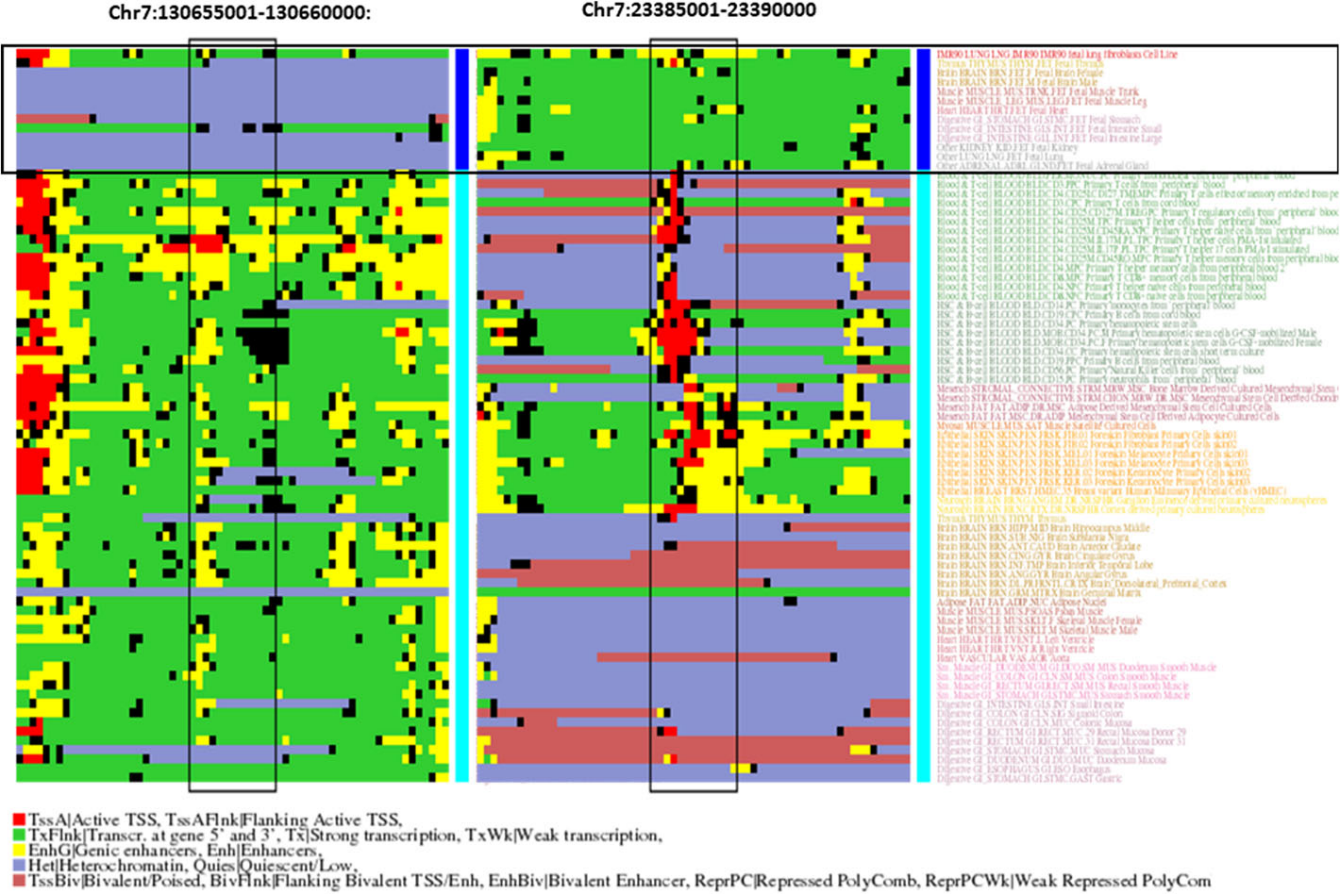
Example of two genomic regions with an epigenetic pattern related to fetal samples. Same representation as in Fig. 2 for fetal samples (dark blue) vs. others (light blue). By the Roadmap coloring of the samples it is evident that the “fetal” set contains now a variety of (fetal) tissues/organs

The region with the highest score is Chr7:23385001–23,390,000). Its epigenetic profile shows that it is being transcribed in the fetal samples while heterochromatized in most of the others (Fig. 3). This region overlaps with intronic and exonic regions of gene IGF2BP3, an insulin-like growth factor 2 mRNA binding protein that repress the translation of that protein during late development. The second window with highest score is Chr2:11530001–11,535,000, which partially overlaps with the long non-coding RNA LINC00570, of unknown function. This region has the “enhancer” state in fetal samples and a mixture of heterochromatin and transcription in the others. The next region in the list, in chromosome 8, show a similar epigenetic pattern and there are not annotated genes on it. The fourth high scoring window is again in chromosome 7 (130655001–130,660,000) and has an epigenetic behavior opposed to the first one discussed above: it is heterochromatinized in the fetal samples while transcribing in most of the others (Fig. 3). This window overlaps with a long non-coding RNA induced by p53 (LOC378805). The whole list of high scoring windows, split by chromosomes is available as Additional file 2.

The first and second genes commented above (IGF2BP3 and LINC00570) also are those with the highest concentration of high-scoring windows (11 and 21 respectively), followed by IGF2BP1 (functionally similar to IGF2BP3) with 7 windows.

Regarding the patterns of epigenetic change between fetal and adult tissues in these windows, Fig. 5 shows a plainer distribution than in the other two datasets, indicating that the preference for certain transitions is not so clear. Nevertheless, the three most frequent transitions are those from transcription, enhancer and bivalent states in fetal to heterochromatin in adult, followed by two patterns representing a somehow opposite transition: transcription in adult vs. heterochromatin/bivalent in fetal. The GO terms associated to the windows within these five subsets are related, respectively, to *none*, extracellular matrix, neuron biogenesis/cell cycle, nucleotide metabolic processes and ossification/immune response (Additional file 4).

### Cancer samples

In this case we detected 345 regions in the human genome, with chromosomes 1 and 2 inclosing the largest number of them (33 each) (Additional file 2).

These regions overlap with 185 annotated genes. Ninety-six regions (28%) do not overlap with any gene. This set of genes is enriched in biological processes clearly related to cancer, such as “cell cycle”, “mitotic cell cycle”, “regulation of transcription involved in cell fate” and “microtubule-based movement” (Table 1). The only enriched GO_CC term is “kinesin complex”. As with the other datasets, the whole list of enriched terms can be found in the Additional file 3, and their REVIGO graphical summary in Additional file 1: Figure S5.

There are five regions with the highest score (0.994) and a *p*-value of 0.0, two of them in chromosome 7, and the others in chromosomes 6 and 4. All overlap with genes known or potentially involved in cancer: JAZF1 (transcriptional repressor for which chromosomal aberrations involving it are associated with stromal tumors), PLXN4A (involved in cytoskeleton remodeling), SCML4 (involved in a complex required to maintain the transcriptionally repressive state of homeotic genes throughout development) and RBPJ (a transcriptional regulator important in the Notch signaling pathway), respectively.

**Fig. 4:**
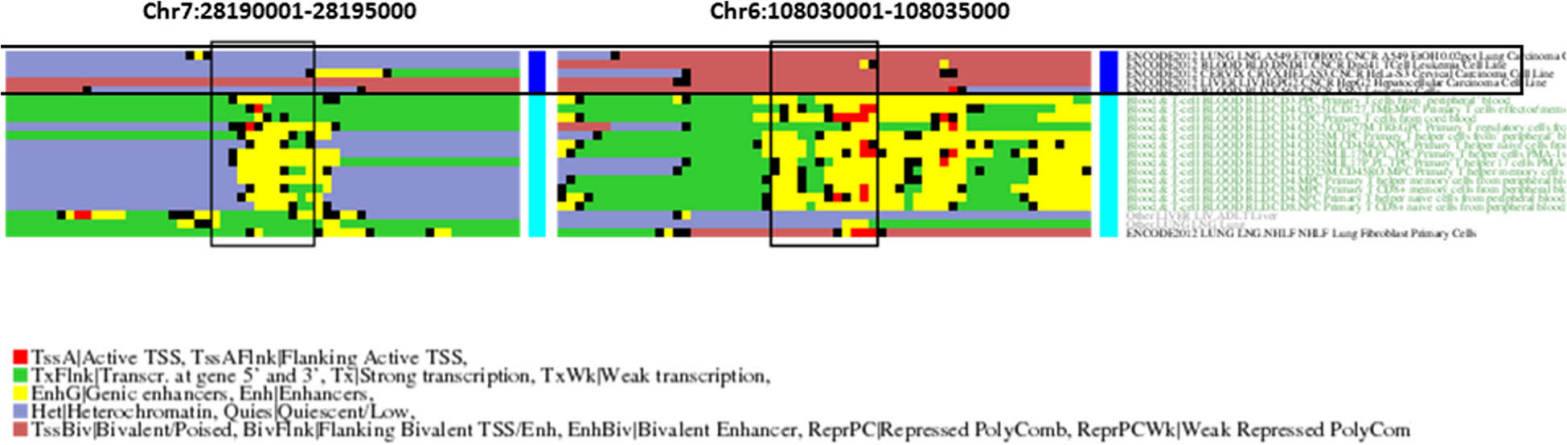
Example of two genomic regions with an epigenetic pattern related to cancer. Same representation as in Figs. 2 and 3 for cancer samples (dark blue) vs. healthy tissues (light blue)

Figure 4 shows the epigenetic profiles for two of these regions: those in chromosomes 7 and 6. In both cases the epigenetic patterns would be in agreement with the roles of the genes coded there. For example, the region in chromosome 7 (gene JAZF1) is active in healthy tissues, hence producing the transcriptional repressor, while in cancer tissues it is heterochromatized and, consequently, the repressor is not being transcribed.

**Fig. 5:**
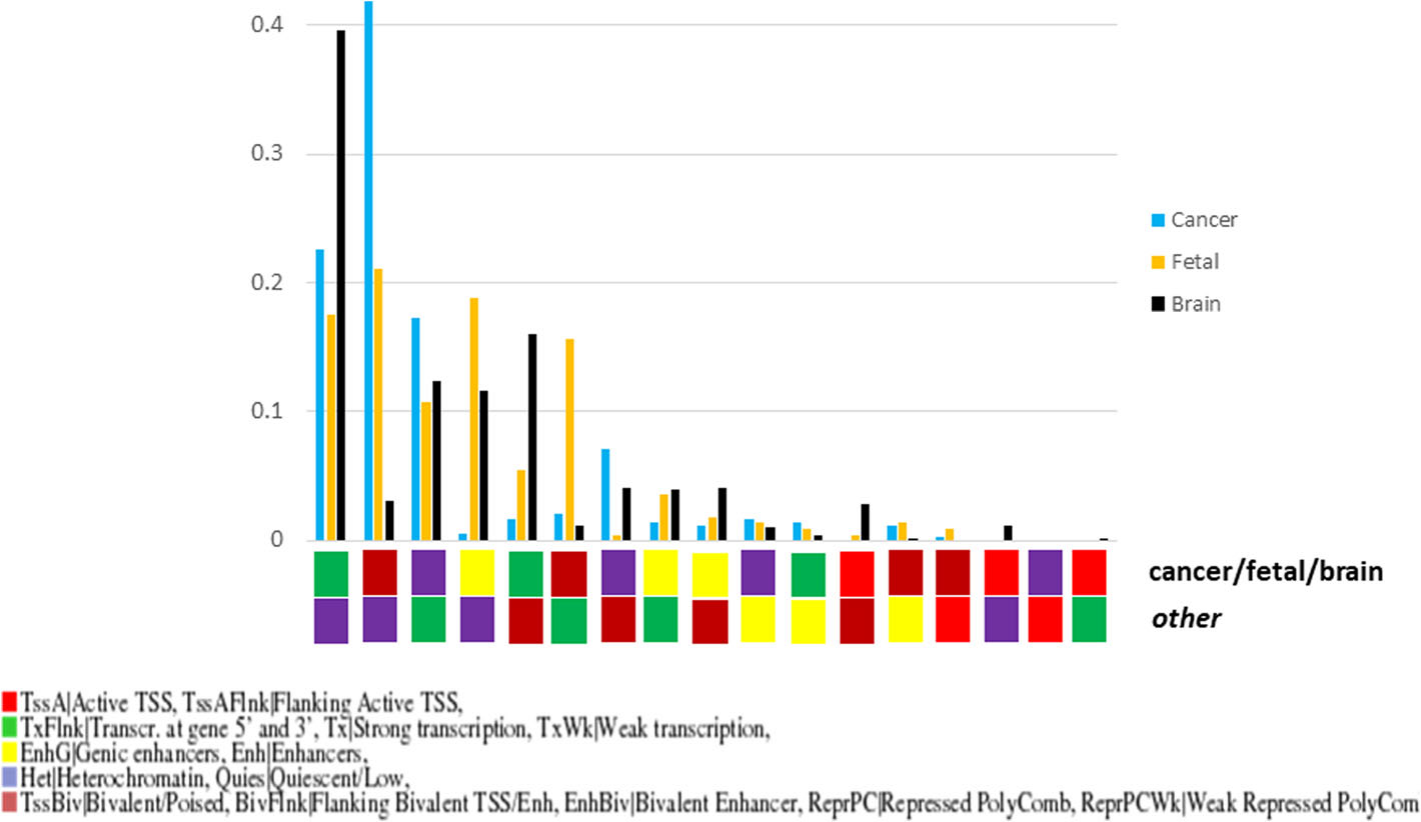
Distribution of patterns of epigenetic change in the genomic segments detected for the three datasets. In the X axis, the patterns of epigenetic change are represented. The top box shows the majority epigenetic state for the segment in the samples of a given class (brain, fetal or cancer), and the box below that of the complementary set. The Y axis represents the proportion of detected genomic segments showing that particular pattern of change for the three datasets. For example, 20% of the detected segments in the “fetal” dataset (gold) are in “enhancer” state (yellow) in the fetal samples while “heterochromatized”(purple) in the adult samples

In spite of these particular examples, in the cancer-related genomic segments, globally the most frequent patterns of epigenetic change between cancer and healthy tissues are transcription (cancer) / heterochromatin (healthy) and bivalent (c) / heterochromatin (h) (Fig. 5). The first pattern is differentially enriched in terms related to neuron action potential, while there are no differentially enriched terms for the second (Additional file 4).

As with the other examples, the concentration of 5000 bp windows in a given gene is also informative. The gene with the highest accumulation of windows is LYST (14 windows), a lysosomal trafficking regulator whose relationship with cancer is not evident. The next one (10 windows) is KIF4A, a kinesin involved in maintaining chromosome integrity during mitosis and organizing the mitotic spindle prior to cytokinesis.

## Discussion

While the DNA sequence of the human genome is virtually the same across all developmental stages, cell types and tissues, its epigenetic state varies, largely contributing to this cellular phenotypic diversity. Consequently, the massive epigenomic data that are being generated are expected to enclose a lot of new information on chromatin dynamics during development, cell differentiation and disease. Resembling what happened with genomic data, many computational approaches are being developed to extract that information from the massive epigenomic datasets.

The approach we present here allows to automatically detect functional regions in the genome, as those with patterns of inter-sample epigenetic variations correlated with an external classification of these samples in an arbitrary number of groups. Not only the regions are detected but it is also possible to get insight into their role by looking into more detail on their particular variation of epigenetic patterns (e.g. active in a group of samples while inactive in others, enhancer, etc.).

Even if it is not an exhaustive test, the results of the “downsampling” experiment with the brain dataset seems to indicate that the method is quite robust to missing data (i.e. lack of epigenomes). The results with the cancer dataset, consisting only in 21 epigenomes, points into the same direction. In any case, we expect the epigenomic data to increase substantially in the future.

The same set of samples can be used to extract different types of functional regions as long as they can be classified in different ways, as we showed here with three orthogonal classifications of the same Roadmap Consortium epigenomes. In this sense the method can be regarded as “supervised”, as the sample classification is provided by the user. This is a fundamental difference with another approach for detecting genome regions with a particular epigenetic pattern, ChromDet [22]. ChromDet is “unsupervised”, in the sense that it detects the regions better reflecting the main tendencies in the set of epigenomes given as input. Another difference is that, due to its computational cost, the method presented here can be applied to large sets of epigenomes and to exhaustively scan the whole genome in detail (i.e. with small windows). In spite of being suitable for scanning whole genomes, epigenetic information for the whole human genome is not strictly required for this approach, and it could work with epigenetic data for a limited region (across a set of samples), while ChromDet requires whole (or very large) epigenomes for extracting their main tendencies. The recently developed ChromSwitch method [19] is also intended to locate regions where certain epigenetic marks change drastically between two sets of samples and, as in our case, it can also work with epigenomic information for a limited region. Apart from using a totally different methodology, the two main differences with the approach described here are its limitation to two groups of samples and the fact that it uses the raw quantitative epigenetic marks as input, instead of a vocabulary of states. Consequently, all these approaches complement each other in the search for chromosomal regions with informative epigenetic profiles.

Although we focused our large scale (enrichment-based) evaluation on regions containing genes, the method is intended to scan the whole genome and detect functional non-coding regions such as enhancers, regulatory regions, etc. Indeed, many of the regions we detected do not overlap with genes. A more detailed inspection of them will be required to assess whether they could be functionally or not (e.g. presence of binding sites for transcription factors, functional RNAs, etc.)

The sliding-window approach allows scanning the genome with different window sizes and moving them at different steps so that, for example, once a large segment of interest is found, smaller windows could be used to scan it in more detail.

As with many genomic studies, this approach still has the problem of distinguishing correlation from causation [31]. It is difficult to assess whether the epigenetic behavior of the genomic regions we are detecting related to a given phenomenon is a cause of that phenomenon or just a consequence/marker of it. Further inspection of the actual epigenetic patterns could shed light into this. In the three datasets we found examples of windows with both between-classes transitions: transcription > heterochromatin and the other way around (heterochromatin > transcription), and in some cases these transitions make sense in the light of the molecular processes associated to the genes of the corresponding windows. In case we can detect regions whose epigenetic variation is causative for the studied phenomenon/disease, these results would be useful for a field that is receiving increasing attention, “epigenome editing” [31, 32]: i.e. modify epigenetics by changing the DNA motifs responsible for it (e.g. with CRISPR/Cas9) or by manipulating the editing enzymes. In the second case, when the detected epigenetic pattern is not causative of the phenomenon but just a reflection of it, it could be used as a marker to detect/monitor it.

## Conclusions

The simple and scalable methodology presented here is able to detect functional genomic regions related to a given phenomenon using epigenomic information.

Paralleling what happened in genomics, we foresee a large accumulation of epigenomes in the future, even at the single-cell level [33]. So, methods such as that presented here, for mining that massive information and translating it into knowledge, would be valuable.

## Additional files

Additional file 1: Additional figures and data. Figures S1, S2, S3, S4 and S5. Epigenome classifications for the three experiments. Detailed description of all epigenomes used. (DOCX 425 kb)

Additional file 2: Full list of windows in all chromosomes for the three experiments (in plain text and html format). The lists include the detailed epigenetic profiles for the windows as well as links to inspect these in a genome browser. (ZIP 973 kb)

Additional file 3: Full lists of GO enriched terms (BP: biological processes, and CC: cellular compartment) for the three experiments, including the genes overlapping with the detected windows within each GO term. (ZIP 33 kb)

Additional file 4: Full lists of GO enriched terms (BP: biological processes, and CC: cellular compartment) for the subsets of windows showing particular patterns of epigenetic change (Fig. 5). (ZIP 72 kb)
