# Supplementary material for: Automatic detection of genomic regions with informative epigenetic patterns
Source: BMC Genomics. 2018 Nov 28;19:847. doi: 10.1186/s12864-018-5286-5 (PMC6264639; doi:10.1186/s12864-018-5286-5)
Supplement: Supplementary file 1 — Additional figures and data. Figures S1, S2, S3, S4 and S5. Epigenome classifications for the three experiments. Detailed description of all epigenomes used. (DOCX 425 kb) [file 12864_2018_5286_MOESM1_ESM.docx]

Automatic detection of genomic regions with informative epigenetic patterns

## Additional Figures and Data

Florencio Pazos*, Adrian Garcia-Moreno & Juan C. Oliveros


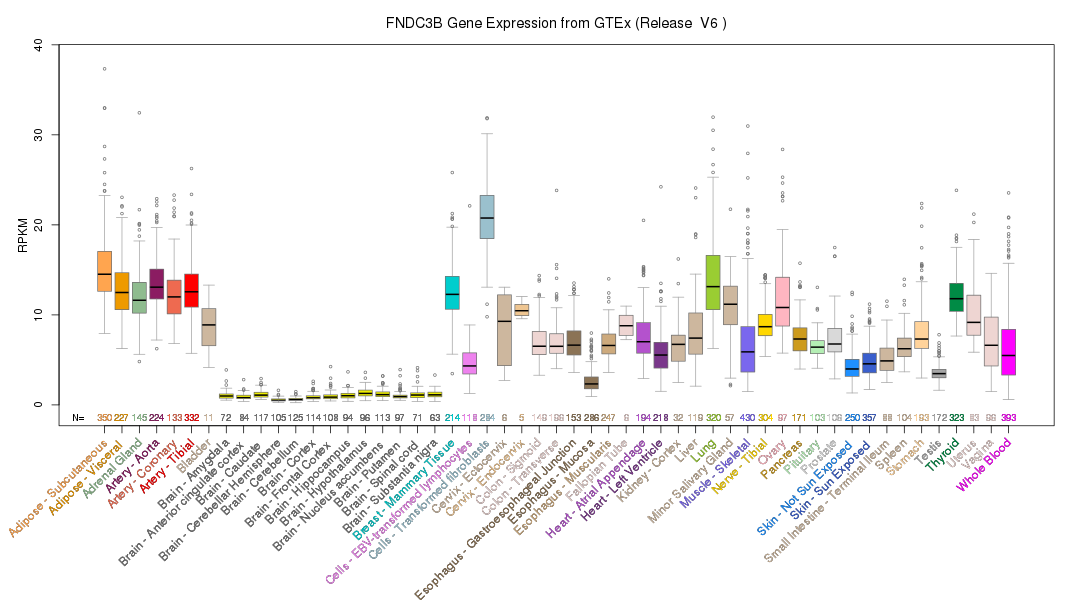


**Figure S1.** Tissue expression of gene FNDC3B (a fribonectin type III containing protein) as reported in GTEx.


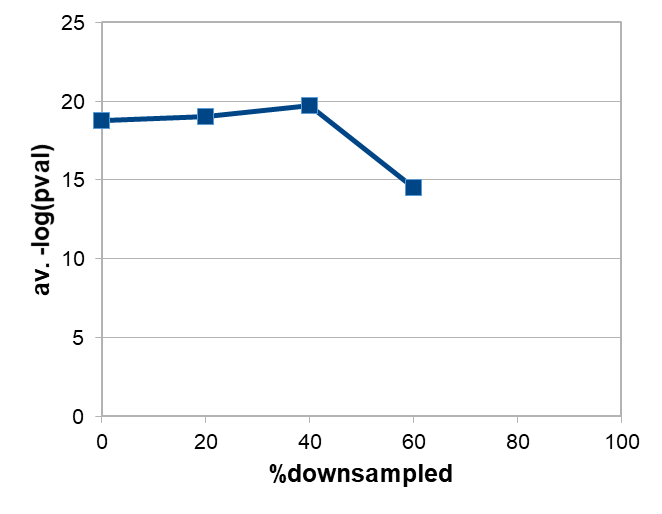


**Figure S2.** Effect of epigenome removal on the method’s performance on the “brain” test set. The X axis represent the proportion of genomes removed (each point is the average of 5 trials) and the Y axis the “performance”, quantified as the minus logarithm of the enrichment score of the positive terms (related to “brain”). For 80% we either ended up without any brain sample in the final set or no terms become enriched.


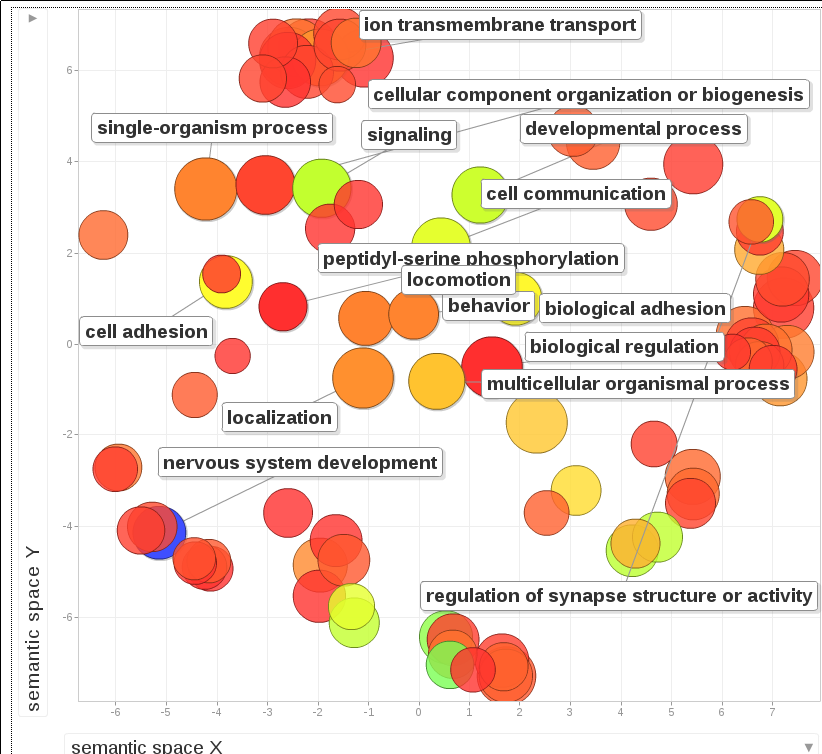


**Figure S3.** REVIGO representation of the enriched GO:BP terms for the brain dataset. REVIGO was run with default parameters except similarity_index>=0.5. The whole list of terms is available in the Additional File 3.


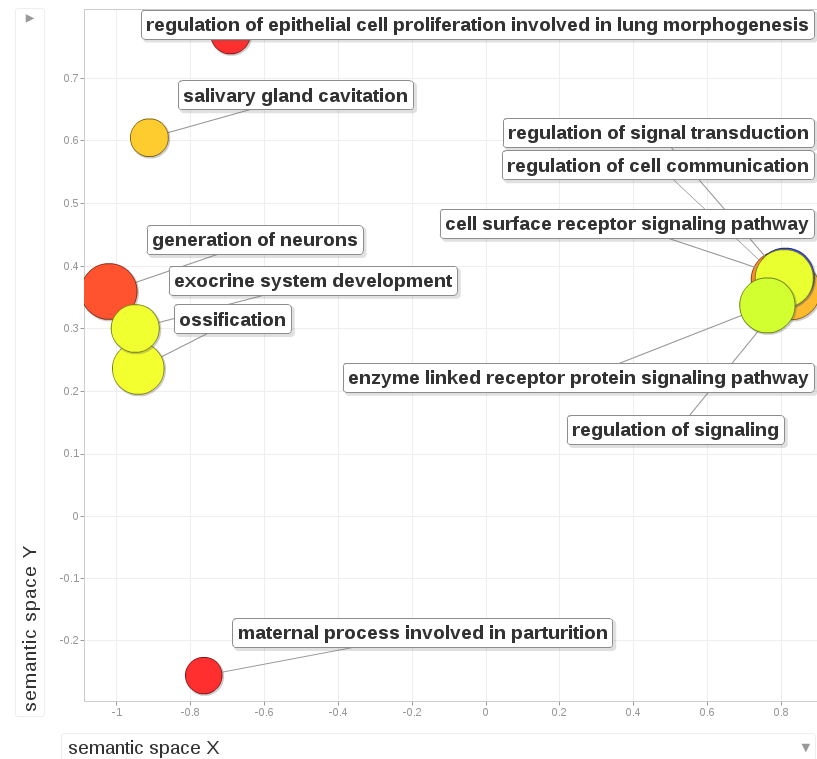


**Figure S4.** REVIGO representation of the enriched GO:BP terms for the fetal dataset. REVIGO was run with default parameters except similarity_index>=0.5. The whole list of terms is available in the Additional File 3.


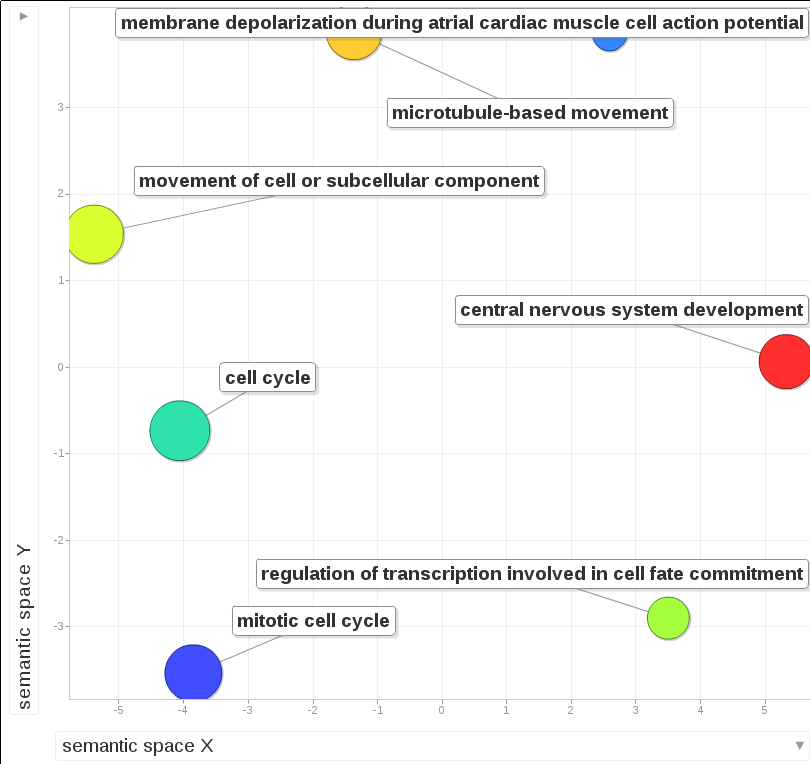


**Figure S5.** REVIGO representation of the enriched GO:BP terms for the cancer dataset. REVIGO was run with default parameters except similarity_index>=0.5. The whole list of terms is available in the Additional File 3.

**NIH Roadmap epigenomes used**

**Epigenome ID (EID) COLOR GROUP ANATOMY Epigenome Mnemonic Standardized Epigenome name**

E017 #E41A1C IMR90 LUNG LNG.IMR90 IMR90 fetal lung fibroblasts Cell Line

E002 #924965 ESC ESC ESC.WA7 ES-WA7 Cells

E008 #924965 ESC ESC ESC.H9 H9 Cells

E001 #924965 ESC ESC ESC.I3 ES-I3 Cells

E015 #924965 ESC ESC ESC.HUES6 HUES6 Cells

E014 #924965 ESC ESC ESC.HUES48 HUES48 Cells

E016 #924965 ESC ESC ESC.HUES64 HUES64 Cells

E003 #924965 ESC ESC ESC.H1 H1 Cells

E024 #924965 ESC ESC ESC.4STAR ES-UCSF4 Cells

E020 #69608A iPSC IPSC IPSC.20B iPS-20b Cells

E019 #69608A iPSC IPSC IPSC.18 iPS-18 Cells

E018 #69608A iPSC IPSC IPSC.15b iPS-15b Cells

E021 #69608A iPSC IPSC IPSC.DF.6.9 iPS DF 6.9 Cells

E022 #69608A iPSC IPSC IPSC.DF.19.11 iPS DF 19.11 Cells

E007 #4178AE ES-deriv ESC_DERIVED ESDR.H1.NEUR.PROG H1 Derived Neuronal Progenitor Cultured Cells

E009 #4178AE ES-deriv ESC_DERIVED ESDR.H9.NEUR.PROG H9 Derived Neuronal Progenitor Cultured Cells

E010 #4178AE ES-deriv ESC_DERIVED ESDR.H9.NEUR H9 Derived Neuron Cultured Cells

E013 #4178AE ES-deriv ESC_DERIVED ESDR.CD56.MESO hESC Derived CD56+ Mesoderm Cultured Cells

E012 #4178AE ES-deriv ESC_DERIVED ESDR.CD56.ECTO hESC Derived CD56+ Ectoderm Cultured Cells

E011 #4178AE ES-deriv ESC_DERIVED ESDR.CD184.ENDO hESC Derived CD184+ Endoderm Cultured Cells

E004 #4178AE ES-deriv ESC_DERIVED ESDR.H1.BMP4.MESO H1 BMP4 Derived Mesendoderm Cultured Cells

E005 #4178AE ES-deriv ESC_DERIVED ESDR.H1.BMP4.TROP H1 BMP4 Derived Trophoblast Cultured Cells

E006 #4178AE ES-deriv ESC_DERIVED ESDR.H1.MSC H1 Derived Mesenchymal Stem Cells

E062 #55A354 Blood & T-cell BLOOD BLD.PER.MONUC.PC Primary mononuclear cells fromÂ peripheralÂ blood

E034 #55A354 Blood & T-cell BLOOD BLD.CD3.PPC Primary T cells fromÂ peripheralÂ blood

E045 #55A354 Blood & T-cell BLOOD BLD.CD4.CD25I.CD127.TMEMPC Primary T cells effector/memory enriched from peripheral blood

E033 #55A354 Blood & T-cell BLOOD BLD.CD3.CPC Primary T cells from cord blood

E044 #55A354 Blood & T-cell BLOOD BLD.CD4.CD25.CD127M.TREGPC Primary T regulatory cells fromÂ peripheralÂ blood

E043 #55A354 Blood & T-cell BLOOD BLD.CD4.CD25M.TPC Primary T helper cells fromÂ peripheralÂ blood

E039 #55A354 Blood & T-cell BLOOD BLD.CD4.CD25M.CD45RA.NPC Primary T helper naive cells fromÂ peripheralÂ blood

E041 #55A354 Blood & T-cell BLOOD BLD.CD4.CD25M.IL17M.PL.TPC Primary T helper cells PMA-I stimulated

E042 #55A354 Blood & T-cell BLOOD BLD.CD4.CD25M.IL17P.PL.TPC Primary T helper 17 cells PMA-I stimulated

E040 #55A354 Blood & T-cell BLOOD BLD.CD4.CD25M.CD45RO.MPC Primary T helper memory cells from peripheral blood 1

E037 #55A354 Blood & T-cell BLOOD BLD.CD4.MPC Primary T helper memory cells from peripheral blood 2

E048 #55A354 Blood & T-cell BLOOD BLD.CD8.MPC Primary T CD8+ memory cells from peripheral blood

E038 #55A354 Blood & T-cell BLOOD BLD.CD4.NPC Primary T helper naive cells from peripheral blood

E047 #55A354 Blood & T-cell BLOOD BLD.CD8.NPC Primary T CD8+ naive cells from peripheral blood

E029 #678C69 HSC & B-cell BLOOD BLD.CD14.PC Primary monocytes fromÂ peripheralÂ blood

E031 #678C69 HSC & B-cell BLOOD BLD.CD19.CPC Primary B cells from cord blood

E035 #678C69 HSC & B-cell BLOOD BLD.CD34.PC Primary hematopoietic stem cells

E051 #678C69 HSC & B-cell BLOOD BLD.MOB.CD34.PC.M Primary hematopoietic stem cells G-CSF-mobilized Male

E050 #678C69 HSC & B-cell BLOOD BLD.MOB.CD34.PC.F Primary hematopoietic stem cells G-CSF-mobilized Female

E036 #678C69 HSC & B-cell BLOOD BLD.CD34.CC Primary hematopoietic stem cells short term culture

E032 #678C69 HSC & B-cell BLOOD BLD.CD19.PPC Primary B cells from peripheral blood

E046 #678C69 HSC & B-cell BLOOD BLD.CD56.PC Primary Natural Killer cells fromÂ peripheralÂ blood

E030 #678C69 HSC & B-cell BLOOD BLD.CD15.PC Primary neutrophils fromÂ peripheralÂ blood

E026 #B65C73 Mesench STROMAL_CONNECTIVE STRM.MRW.MSC Bone Marrow Derived Cultured Mesenchymal Stem Cells

E049 #B65C73 Mesench STROMAL_CONNECTIVE STRM.CHON.MRW.DR.MSC Mesenchymal Stem Cell Derived Chondrocyte Cultured Cells

E025 #B65C73 Mesench FAT FAT.ADIP.DR.MSC Adipose Derived Mesenchymal Stem Cell Cultured Cells

E023 #B65C73 Mesench FAT FAT.MSC.DR.ADIP Mesenchymal Stem Cell Derived Adipocyte Cultured Cells

E052 #E67326 Myosat MUSCLE MUS.SAT Muscle Satellite Cultured Cells

E055 #FF9D0C Epithelial SKIN SKIN.PEN.FRSK.FIB.01 Foreskin Fibroblast Primary Cells skin01

E056 #FF9D0C Epithelial SKIN SKIN.PEN.FRSK.FIB.02 Foreskin Fibroblast Primary Cells skin02

E059 #FF9D0C Epithelial SKIN SKIN.PEN.FRSK.MEL.01 Foreskin Melanocyte Primary Cells skin01

E061 #FF9D0C Epithelial SKIN SKIN.PEN.FRSK.MEL.03 Foreskin Melanocyte Primary Cells skin03

E057 #FF9D0C Epithelial SKIN SKIN.PEN.FRSK.KER.02 Foreskin Keratinocyte Primary Cells skin02

E058 #FF9D0C Epithelial SKIN SKIN.PEN.FRSK.KER.03 Foreskin Keratinocyte Primary Cells skin03

E028 #FF9D0C Epithelial BREAST BRST.HMEC.35 Breast variant Human Mammary Epithelial Cells (vHMEC)

E027 #FF9D0C Epithelial BREAST BRST.MYO Breast Myoepithelial Primary Cells

E054 #FFD924 Neurosph BRAIN BRN.GANGEM.DR.NRSPHR Ganglion Eminence derived primary cultured neurospheres

E053 #FFD924 Neurosph BRAIN BRN.CRTX.DR.NRSPHR Cortex derived primary cultured neurospheres

E112 #DAB92E Thymus THYMUS THYM Thymus

E093 #DAB92E Thymus THYMUS THYM.FET Fetal Thymus

E071 #C5912B Brain BRAIN BRN.HIPP.MID Brain Hippocampus Middle

E074 #C5912B Brain BRAIN BRN.SUB.NIG Brain Substantia Nigra

E068 #C5912B Brain BRAIN BRN.ANT.CAUD Brain Anterior Caudate

E069 #C5912B Brain BRAIN BRN.CING.GYR Brain Cingulate Gyrus

E072 #C5912B Brain BRAIN BRN.INF.TMP Brain Inferior Temporal Lobe

E067 #C5912B Brain BRAIN BRN.ANG.GYR Brain Angular Gyrus

E073 #C5912B Brain BRAIN BRN.DL.PRFRNTL.CRTX Brain_Dorsolateral_Prefrontal_Cortex

E070 #C5912B Brain BRAIN BRN.GRM.MTRX Brain Germinal Matrix

E082 #C5912B Brain BRAIN BRN.FET.F Fetal Brain Female

E081 #C5912B Brain BRAIN BRN.FET.M Fetal Brain Male

E063 #AF5B39 Adipose FAT FAT.ADIP.NUC Adipose Nuclei

E100 #C2655D Muscle MUSCLE MUS.PSOAS Psoas Muscle

E108 #C2655D Muscle MUSCLE MUS.SKLT.F Skeletal Muscle Female

E107 #C2655D Muscle MUSCLE MUS.SKLT.M Skeletal Muscle Male

E089 #C2655D Muscle MUSCLE MUS.TRNK.FET Fetal Muscle Trunk

E090 #C2655D Muscle MUSCLE_LEG MUS.LEG.FET Fetal Muscle Leg

E083 #D56F80 Heart HEART HRT.FET Fetal Heart

E104 #D56F80 Heart HEART HRT.ATR.R Right Atrium

E095 #D56F80 Heart HEART HRT.VENT.L Left Ventricle

E105 #D56F80 Heart HEART HRT.VNT.R Right Ventricle

E065 #D56F80 Heart VASCULAR VAS.AOR Aorta

E078 #F182BC Sm. Muscle GI_DUODENUM GI.DUO.SM.MUS Duodenum Smooth Muscle

E076 #F182BC Sm. Muscle GI_COLON GI.CLN.SM.MUS Colon Smooth Muscle

E103 #F182BC Sm. Muscle GI_RECTUM GI.RECT.SM.MUS Rectal Smooth Muscle

E111 #F182BC Sm. Muscle GI_STOMACH GI.STMC.MUS Stomach Smooth Muscle

E092 #C58DAA Digestive GI_STOMACH GI.STMC.FET Fetal Stomach

E085 #C58DAA Digestive GI_INTESTINE GI.S.INT.FET Fetal Intestine Small

E084 #C58DAA Digestive GI_INTESTINE GI.L.INT.FET Fetal Intestine Large

E109 #C58DAA Digestive GI_INTESTINE GI.S.INT Small Intestine

E106 #C58DAA Digestive GI_COLON GI.CLN.SIG Sigmoid Colon

E075 #C58DAA Digestive GI_COLON GI.CLN.MUC Colonic Mucosa

E101 #C58DAA Digestive GI_RECTUM GI.RECT.MUC.29 Rectal Mucosa Donor 29

E102 #C58DAA Digestive GI_RECTUM GI.RECT.MUC.31 Rectal Mucosa Donor 31

E110 #C58DAA Digestive GI_STOMACH GI.STMC.MUC Stomach Mucosa

E077 #C58DAA Digestive GI_DUODENUM GI.DUO.MUC Duodenum Mucosa

E079 #C58DAA Digestive GI_ESOPHAGUS GI.ESO Esophagus

E094 #C58DAA Digestive GI_STOMACH GI.STMC.GAST Gastric

E099 #999999 Other PLACENTA PLCNT.AMN Placenta Amnion

E086 #999999 Other KIDNEY KID.FET Fetal Kidney

E088 #999999 Other LUNG LNG.FET Fetal Lung

E097 #999999 Other OVARY OVRY Ovary

E087 #999999 Other PANCREAS PANC.ISLT Pancreatic Islets

E080 #999999 Other ADRENAL ADRL.GLND.FET Fetal Adrenal Gland

E091 #999999 Other PLACENTA PLCNT.FET Placenta

E066 #999999 Other LIVER LIV.ADLT Liver

E098 #999999 Other PANCREAS PANC Pancreas

E096 #999999 Other LUNG LNG Lung

E113 #999999 Other SPLEEN SPLN Spleen

E114 #000000 ENCODE2012 LUNG LNG.A549.ETOH002.CNCR A549 EtOH 0.02pct Lung Carcinoma Cell Line

E115 #000000 ENCODE2012 BLOOD BLD.DND41.CNCR Dnd41 TCell Leukemia Cell Line

E116 #000000 ENCODE2012 BLOOD BLD.GM12878 GM12878 Lymphoblastoid Cells

E117 #000000 ENCODE2012 CERVIX CRVX.HELAS3.CNCR HeLa-S3 Cervical Carcinoma Cell Line

E118 #000000 ENCODE2012 LIVER LIV.HEPG2.CNCR HepG2 Hepatocellular Carcinoma Cell Line

E119 #000000 ENCODE2012 BREAST BRST.HMEC HMEC Mammary Epithelial Primary Cells

E120 #000000 ENCODE2012 MUSCLE MUS.HSMM HSMM Skeletal Muscle Myoblasts Cells

E121 #000000 ENCODE2012 MUSCLE MUS.HSMMT HSMM cell derived Skeletal Muscle Myotubes Cells

E122 #000000 ENCODE2012 VASCULAR VAS.HUVEC HUVEC Umbilical Vein Endothelial Primary Cells

E123 #000000 ENCODE2012 BLOOD BLD.K562.CNCR K562 Leukemia Cells

E124 #000000 ENCODE2012 BLOOD BLD.CD14.MONO Monocytes-CD14+ RO01746 Primary Cells

E125 #000000 ENCODE2012 BRAIN BRN.NHA NH-A Astrocytes Primary Cells

E126 #000000 ENCODE2012 SKIN SKIN.NHDFAD NHDF-Ad Adult Dermal Fibroblast Primary Cells

E127 #000000 ENCODE2012 SKIN SKIN.NHEK NHEK-Epidermal Keratinocyte Primary Cells

E128 #000000 ENCODE2012 LUNG LNG.NHLF NHLF Lung Fibroblast Primary Cells

E129 #000000 ENCODE2012 BONE BONE.OSTEO Osteoblast Primary Cells

**Epigenomes used in the “brain” test. “2”: brain, “1”: other.**

E071_15_coreMarks_mnemonics.bed 2

E074_15_coreMarks_mnemonics.bed 2

E068_15_coreMarks_mnemonics.bed 2

E069_15_coreMarks_mnemonics.bed 2

E072_15_coreMarks_mnemonics.bed 2

E067_15_coreMarks_mnemonics.bed 2

E073_15_coreMarks_mnemonics.bed 2

E070_15_coreMarks_mnemonics.bed 2

E082_15_coreMarks_mnemonics.bed 2

E081_15_coreMarks_mnemonics.bed 2

E062_15_coreMarks_mnemonics.bed 1

E034_15_coreMarks_mnemonics.bed 1

E045_15_coreMarks_mnemonics.bed 1

E033_15_coreMarks_mnemonics.bed 1

E044_15_coreMarks_mnemonics.bed 1

E043_15_coreMarks_mnemonics.bed 1

E039_15_coreMarks_mnemonics.bed 1

E041_15_coreMarks_mnemonics.bed 1

E042_15_coreMarks_mnemonics.bed 1

E040_15_coreMarks_mnemonics.bed 1

E037_15_coreMarks_mnemonics.bed 1

E048_15_coreMarks_mnemonics.bed 1

E038_15_coreMarks_mnemonics.bed 1

E047_15_coreMarks_mnemonics.bed 1

E029_15_coreMarks_mnemonics.bed 1

E031_15_coreMarks_mnemonics.bed 1

E035_15_coreMarks_mnemonics.bed 1

E051_15_coreMarks_mnemonics.bed 1

E050_15_coreMarks_mnemonics.bed 1

E036_15_coreMarks_mnemonics.bed 1

E032_15_coreMarks_mnemonics.bed 1

E046_15_coreMarks_mnemonics.bed 1

E030_15_coreMarks_mnemonics.bed 1

E026_15_coreMarks_mnemonics.bed 1

E049_15_coreMarks_mnemonics.bed 1

E025_15_coreMarks_mnemonics.bed 1

E023_15_coreMarks_mnemonics.bed 1

E052_15_coreMarks_mnemonics.bed 1

E055_15_coreMarks_mnemonics.bed 1

E056_15_coreMarks_mnemonics.bed 1

E059_15_coreMarks_mnemonics.bed 1

E061_15_coreMarks_mnemonics.bed 1

E057_15_coreMarks_mnemonics.bed 1

E058_15_coreMarks_mnemonics.bed 1

E028_15_coreMarks_mnemonics.bed 1

E054_15_coreMarks_mnemonics.bed 1

E053_15_coreMarks_mnemonics.bed 1

E112_15_coreMarks_mnemonics.bed 1

E093_15_coreMarks_mnemonics.bed 1

E063_15_coreMarks_mnemonics.bed 1

E100_15_coreMarks_mnemonics.bed 1

E108_15_coreMarks_mnemonics.bed 1

E107_15_coreMarks_mnemonics.bed 1

E089_15_coreMarks_mnemonics.bed 1

E090_15_coreMarks_mnemonics.bed 1

E083_15_coreMarks_mnemonics.bed 1

E095_15_coreMarks_mnemonics.bed 1

E105_15_coreMarks_mnemonics.bed 1

E065_15_coreMarks_mnemonics.bed 1

E078_15_coreMarks_mnemonics.bed 1

E076_15_coreMarks_mnemonics.bed 1

E103_15_coreMarks_mnemonics.bed 1

E111_15_coreMarks_mnemonics.bed 1

E092_15_coreMarks_mnemonics.bed 1

E085_15_coreMarks_mnemonics.bed 1

E084_15_coreMarks_mnemonics.bed 1

E109_15_coreMarks_mnemonics.bed 1

E106_15_coreMarks_mnemonics.bed 1

E075_15_coreMarks_mnemonics.bed 1

E101_15_coreMarks_mnemonics.bed 1

E102_15_coreMarks_mnemonics.bed 1

E110_15_coreMarks_mnemonics.bed 1

E077_15_coreMarks_mnemonics.bed 1

E079_15_coreMarks_mnemonics.bed 1

E094_15_coreMarks_mnemonics.bed 1

**Epigenomes used in the “cancer” test. “1”: cancer, “2”: healthy tissues.**

E114_15_coreMarks_mnemonics.bed 1

E115_15_coreMarks_mnemonics.bed 1

E117_15_coreMarks_mnemonics.bed 1

E118_15_coreMarks_mnemonics.bed 1

E123_15_coreMarks_mnemonics.bed 1

E034_15_coreMarks_mnemonics.bed 2

E045_15_coreMarks_mnemonics.bed 2

E033_15_coreMarks_mnemonics.bed 2

E044_15_coreMarks_mnemonics.bed 2

E043_15_coreMarks_mnemonics.bed 2

E039_15_coreMarks_mnemonics.bed 2

E041_15_coreMarks_mnemonics.bed 2

E042_15_coreMarks_mnemonics.bed 2

E040_15_coreMarks_mnemonics.bed 2

E037_15_coreMarks_mnemonics.bed 2

E048_15_coreMarks_mnemonics.bed 2

E038_15_coreMarks_mnemonics.bed 2

E047_15_coreMarks_mnemonics.bed 2

E066_15_coreMarks_mnemonics.bed 2

E096_15_coreMarks_mnemonics.bed 2

E128_15_coreMarks_mnemonics.bed 2

**Epigenomes used in the “fetal” test. “1”: fetal, “2”: adult.**

E017_15_coreMarks_mnemonics.bed 1

E093_15_coreMarks_mnemonics.bed 1

E082_15_coreMarks_mnemonics.bed 1

E081_15_coreMarks_mnemonics.bed 1

E089_15_coreMarks_mnemonics.bed 1

E090_15_coreMarks_mnemonics.bed 1

E083_15_coreMarks_mnemonics.bed 1

E092_15_coreMarks_mnemonics.bed 1

E085_15_coreMarks_mnemonics.bed 1

E084_15_coreMarks_mnemonics.bed 1

E086_15_coreMarks_mnemonics.bed 1

E088_15_coreMarks_mnemonics.bed 1

E080_15_coreMarks_mnemonics.bed 1

E062_15_coreMarks_mnemonics.bed 2

E034_15_coreMarks_mnemonics.bed 2

E045_15_coreMarks_mnemonics.bed 2

E033_15_coreMarks_mnemonics.bed 2

E044_15_coreMarks_mnemonics.bed 2

E043_15_coreMarks_mnemonics.bed 2

E039_15_coreMarks_mnemonics.bed 2

E041_15_coreMarks_mnemonics.bed 2

E042_15_coreMarks_mnemonics.bed 2

E040_15_coreMarks_mnemonics.bed 2

E037_15_coreMarks_mnemonics.bed 2

E048_15_coreMarks_mnemonics.bed 2

E038_15_coreMarks_mnemonics.bed 2

E047_15_coreMarks_mnemonics.bed 2

E029_15_coreMarks_mnemonics.bed 2

E031_15_coreMarks_mnemonics.bed 2

E035_15_coreMarks_mnemonics.bed 2

E051_15_coreMarks_mnemonics.bed 2

E050_15_coreMarks_mnemonics.bed 2

E036_15_coreMarks_mnemonics.bed 2

E032_15_coreMarks_mnemonics.bed 2

E046_15_coreMarks_mnemonics.bed 2

E030_15_coreMarks_mnemonics.bed 2

E026_15_coreMarks_mnemonics.bed 2

E049_15_coreMarks_mnemonics.bed 2

E025_15_coreMarks_mnemonics.bed 2

E023_15_coreMarks_mnemonics.bed 2

E052_15_coreMarks_mnemonics.bed 2

E055_15_coreMarks_mnemonics.bed 2

E056_15_coreMarks_mnemonics.bed 2

E059_15_coreMarks_mnemonics.bed 2

E061_15_coreMarks_mnemonics.bed 2

E057_15_coreMarks_mnemonics.bed 2

E058_15_coreMarks_mnemonics.bed 2

E028_15_coreMarks_mnemonics.bed 2

E054_15_coreMarks_mnemonics.bed 2

E053_15_coreMarks_mnemonics.bed 2

E112_15_coreMarks_mnemonics.bed 2

E071_15_coreMarks_mnemonics.bed 2

E074_15_coreMarks_mnemonics.bed 2

E068_15_coreMarks_mnemonics.bed 2

E069_15_coreMarks_mnemonics.bed 2

E072_15_coreMarks_mnemonics.bed 2

E067_15_coreMarks_mnemonics.bed 2

E073_15_coreMarks_mnemonics.bed 2

E070_15_coreMarks_mnemonics.bed 2

E063_15_coreMarks_mnemonics.bed 2

E100_15_coreMarks_mnemonics.bed 2

E108_15_coreMarks_mnemonics.bed 2

E107_15_coreMarks_mnemonics.bed 2

E095_15_coreMarks_mnemonics.bed 2

E105_15_coreMarks_mnemonics.bed 2

E065_15_coreMarks_mnemonics.bed 2

E078_15_coreMarks_mnemonics.bed 2

E076_15_coreMarks_mnemonics.bed 2

E103_15_coreMarks_mnemonics.bed 2

E111_15_coreMarks_mnemonics.bed 2

E109_15_coreMarks_mnemonics.bed 2

E106_15_coreMarks_mnemonics.bed 2

E075_15_coreMarks_mnemonics.bed 2

E101_15_coreMarks_mnemonics.bed 2

E102_15_coreMarks_mnemonics.bed 2

E110_15_coreMarks_mnemonics.bed 2

E077_15_coreMarks_mnemonics.bed 2

E079_15_coreMarks_mnemonics.bed 2

E094_15_coreMarks_mnemonics.bed 2
