# Supplementary material for: Automatic detection of genomic regions with informative epigenetic patterns
Source: BMC Genomics. 2018 Nov 28;19:847. doi: 10.1186/s12864-018-5286-5 (PMC6264639; doi:10.1186/s12864-018-5286-5)
Supplement: Supplementary file 2 — Full list of windows in all chromosomes for the three experiments (in plain text and html format). The lists include the detailed epigenetic profiles for the windows as well as links to inspect these in a genome browser. (ZIP 973 kb) [file 12864_2018_5286_MOESM2_ESM.zip › Additional_file_2/brain/HTML/sdp_brain_24_15to5_3_E4.html]

```
# Epicorr 1.19
#
# nsamples= 75  # Nr. of epigenomes (samples)
# wlen= 5000  # Window length (bp)
# step= 5000  # Distance between window inits (bp)
#           => Window 'n' goes from  (n-1)*step+1  to  (n-1)*step+wlen  (bp) 
# Chr_1= 24 (Y) 59432939 bp
# nwin1= 11885 # Nr. of windows for Chr_1
# Chr_2= 24 (Y) 59432939 bp
# nwin2= 11885 # Nr. of windows for Chr_2
# Epigen vocabulary:
# epired= YES # Reduced vocabulary (yes/no)
# state  mnemonic  color  shortname|longname
#   0	-	#000000	(---|---, ZNF/Rpts|ZNF genes & repeats, )
#   1	A	#FF0000	(TssA|Active TSS, TssAFlnk|Flanking Active TSS, )
#   3	5	#32CD32	(TxFlnk|Transcr. at gene 5' and 3', Tx|Strong transcription, TxWk|Weak transcription, )
#   7	E	#FFFF00	(EnhG|Genic enhancers, Enh|Enhancers, )
#   9	h	#8A91D0	(Het|Heterochromatin, Quies|Quiescent/Low, )
#  10	B	#CD5C5C	(TssBiv|Bivalent/Poised, BivFlnk|Flanking Bivalent TSS/Enh, EnhBiv|Bivalent Enhancer, ReprPC|Repressed PolyComb, ReprPCWk|Weak Repressed PolyComb, )
#------------------------------------------------------------
```
