# Supplementary figures and images for: Automatic detection of genomic regions with informative epigenetic patterns
Source: BMC Genomics. 2018 Nov 28;19:847. doi: 10.1186/s12864-018-5286-5 (PMC6264639; doi:10.1186/s12864-018-5286-5)

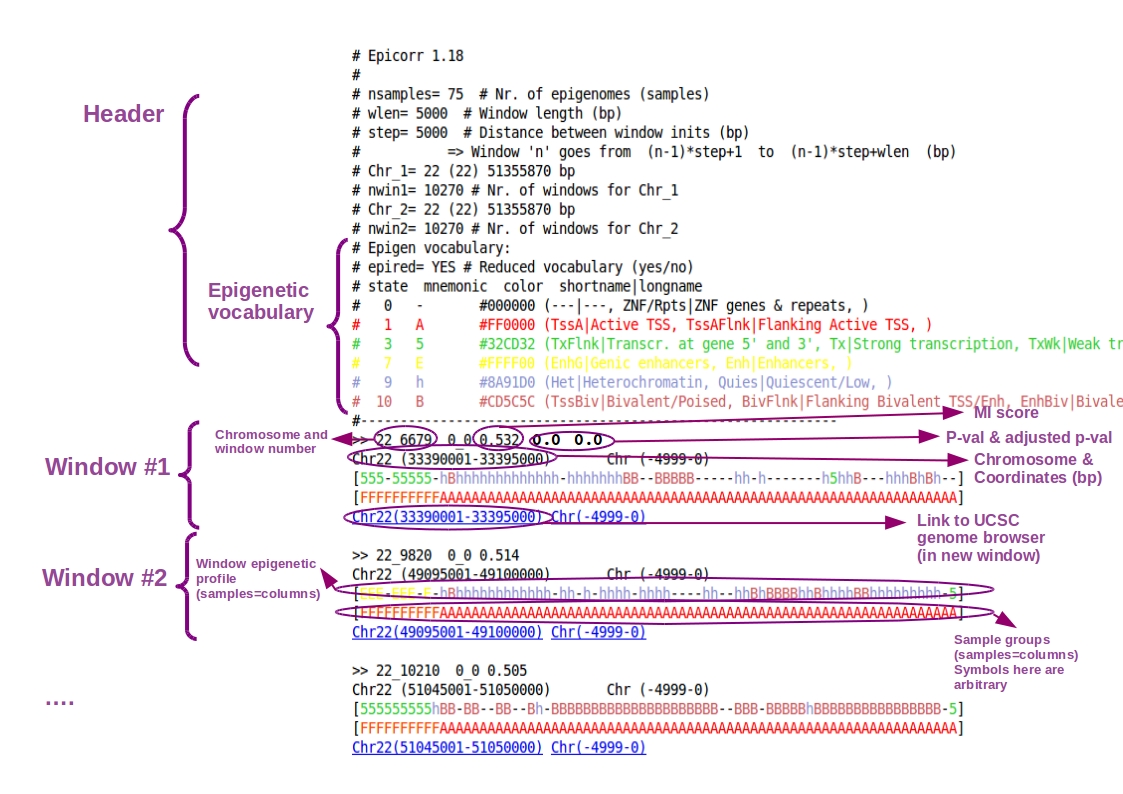

Supplement: Supplementary file 2 — Full list of windows in all chromosomes for the three experiments (in plain text and html format). The lists include the detailed epigenetic profiles for the windows as well as links to inspect these in a genome browser. (ZIP 973 kb) [file 12864_2018_5286_MOESM2_ESM.zip › Additional_file_2/00README.jpg]
